# Trancriptional landscape of *Aspergillus niger* at breaking of conidial dormancy revealed by RNA-sequencing

**DOI:** 10.1186/1471-2164-14-246

**Published:** 2013-04-11

**Authors:** Michaela Novodvorska, Kimran Hayer, Steven T Pullan, Raymond Wilson, Martin J Blythe, Hein Stam, Malcolm Stratford, David B Archer

**Affiliations:** 1 School of Biology, University of Nottingham, University Park, Nottingham, NG7 2RD, UK; 2 Deep Sequencing Facility, Queen's Medical Centre, University of Nottingham, Nottingham, NG7 2UH, UK; 3 DSM Food Specialties, Research and Development, P.O. Box 1, Delft, 2600 MA, The Netherlands

**Keywords:** *Aspergillus niger*, Conidia, Dormancy, Transcriptome, RNA-seq, Antisense transcription

## Abstract

**Background:**

Genome-wide analysis was performed to assess the transcriptional landscape of germinating *A. niger* conidia using both next generation RNA-sequencing and GeneChips. The metabolism of storage compounds during conidial germination was also examined and compared to the transcript levels from associated genes.

**Results:**

The transcriptome of dormant conidia was shown to be highly differentiated from that of germinating conidia and major changes in response to environmental shift occurred within the first hour of germination. The breaking of dormancy was associated with increased transcript levels of genes involved in the biosynthesis of proteins, RNA turnover and respiratory metabolism. Increased transcript levels of genes involved in metabolism of nitrate at the onset of germination implies its use as a source of nitrogen. The transcriptome of dormant conidia contained a significant component of antisense transcripts that changed during germination.

**Conclusion:**

Dormant conidia contained transcripts of genes involved in fermentation, gluconeogenesis and the glyoxylate cycle. The presence of such transcripts in dormant conidia may indicate the generation of energy from non-carbohydrate substrates during starvation-induced conidiation or for maintenance purposes during dormancy. The immediate onset of metabolism of internal storage compounds after the onset of germination, and the presence of transcripts of relevant genes, suggest that conidia are primed for the onset of germination. For some genes, antisense transcription is regulated in the transition from resting conidia to fully active germinants.

## Background

Fungal spores are reproductive structures that are important for both dispersal and survival within harsh environments. Conidia, which are asexual spores, can remain viable for over a year and they begin to germinate as soon as they detect suitable environmental conditions [1]. They possess mechanisms that protect them from ambient stresses. For example, dehydrins are proteins that strongly contribute to resistance against oxidative, osmotic and pH stress and they are highly expressed in dormant conidia [2]. Fungal conidia also produce volatiles that prevent them from untimely germination [3].

The outgrowth of fungal conidia is a key factor in the infection of target organisms by pathogenic fungi as well as in the spoilage of food, emphasizing the need to understand better the transcriptional events during the process of conidial germination. For example, decarboxylation of the food preservative, sorbic acid, is a transcription-dependent and time-dependent activity in developing conidia of *A. niger*[4]. Fungal cells adapt their metabolism in response to environmental nutrient availability and transcripts and proteins present in dormant and germinating conidia reflect, to some extent, the culture conditions [5]. It has also been suggested that dormant conidia exhibit a basal level of metabolism based on detected changes in composition of internal sugars and lipids over storage time [6]. Similarly, dormant ascospores of the budding yeast *Saccharomyces cerevisiae* exhibit essential basal metabolism required for their survival prior to germination [7]. Conidial germination has been studied at the physiological and the molecular levels in various model moulds [1,5,8-10], using proteomic or transcriptomic approaches. The breaking of the dormant state is invariably associated with the processes of water uptake, cell wall remodelling, activation of energy-yielding reactions and biosynthesis of new proteins [1,9]. The presence of oxygen, active mitochondria and a functional respiratory chain are also required [1,11]. *Aspergillus fumigatus* conidia, for example, will not germinate in the absence of water, a degradable carbon source or oxygen [11]. Compatible solutes such as mannitol and trehalose serve as storage carbon sources and give conidia the ability to survive in stress conditions, in elevated temperatures and drought [12,13]. Glycerol and erythritol have been shown to play a role in osmoregulation in *Aspergillus nidulans* and *A. niger* and generate turgor pressure necessary for growth [13,14]. Mannitol and trehalose are known to be degraded during germination [15,16]. Glycerol is the first polyol that disappears during starvation and its biosynthesis occurs during the germination of fungal conidia [13] especially in oxygen-rich environments [12].

*A. niger* has become a useful model in which to study conidial germination due to the availability of published genome sequences [17,18] and well-developed genomic tools. Next generation RNA-sequencing technology (RNA-seq) is a powerful tool for transcriptomic studies. It has been successfully used for improving genome annotations and in investigations of transcriptomes under various conditions in fungi [19,20]. Using this approach, a large number of natural antisense transcripts (NATs) was reported [21]. NATs are RNAs complementary to messenger RNA and they have been identified in many organisms, including fungi, and can regulate gene expression through various mechanisms [21].

In this study, we have used GeneChips to study the transcriptional changes in developing conidia of *A. niger* and showed that most changes occur in the initial period of germination (0-1 h). We then used RNA-seq to study those transcriptional changes in more detail and we have focussed on those transcriptional changes that relate to metabolism and generation of energy.

## Results and discussion

### Functional analysis of differentially-expressed genes

**Figure 1:**
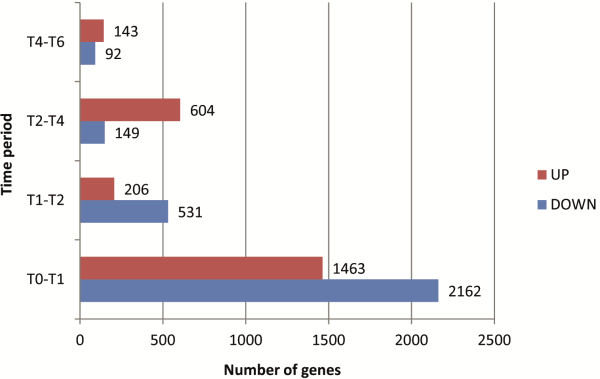
**Numbers of differentially expressed genes.** Numbers of *A. niger* genes having significantly different transcript levels represented by fold-change ≥ 2 between samples from adjacent time-points (0-6 h, T0 – T6) using Gene Chip data. UP – increased transcript levels, DOWN – decreased transcript levels.

GeneChip measurement of transcript levels in freshly harvested dormant conidia (T0) and at 1, 2, 4 and 6 h after inoculation into liquid ACM (T1-T6) showed that transcripts from 20% to 40% of the 14,259 genes represented on the array [17] had a present call at each time point (Additional file 1). Fold-changes in transcript levels were calculated for each time point relative to that directly preceding it (T0-T1, T1-T2, T2-T4, T4-T6) (Additional file 2). Figure 1 shows the number of genes having significantly different transcript levels between samples from adjacent time points and Table 1 lists example genes, based around functionality of encoded proteins in metabolism, that had transcript levels at least two-fold different between each pair of time points. The transcriptional changes occurring during this initial breaking of dormancy were far more wide-ranging than at any other stage within the time course with T1-T2, T2-T4 and T4-T6 transitions.

**Table 1:** Selection of differentially expressed genes at T0-T1 generated by GeneChips and RNA-seq

**Gene**	**Verified/putative function**	**Fc**^**1**^**↑up/↓down**	**RPKM T0**	**RPKM T1**	**RPKM Fc ↑up/↓down**
	**Gluconeogenesis/glyoxylate cycle**				
An04g05300	fructose bisphosphatase (*A. nidulans acuG*)	↓ 31.39	71.07	11.66	↓ 6.09
An11g02550	phosphoenolpyruvate carboxykinase (*A. nidulans acuF*)	↓ 80.62	37.27	2.88	↓ 12.94
An01g09270	isocitrate lyase (*A. nidulans acuD*)	↓ 20.47	627.18	93.74	↓ 6.69
An15g01860	malate synthase (*A. nidulans acuE*)	↓ 40.61	28.52	3.68	↓ 7.75
An12g01990	acyl-CoA synthetase	↓ 2.04	16.85	3.29	↓ 5.12
An07g09190	acyl-CoA synthetase	↓ 6.07	23.8	10.77	↓ 2.20
An08g04990	carnitine acetyl transferase (*A. nidulans facC*)	↓ 20.74	107.81	10.2	↓ 10.56
An08g06580	*facB*, acetate regulatory DNA binding protein	↓ 2.09	16.27	0.99	↓ 16.43
	***Metabolism of alternative carbon sources***				
An13g00480	triacylglycerol lipase	↓ 17.13	10.83	0.36	↓ 30.08
An09g05120	triacylglycerol lipase	↓ 5.09	11.84	0.81	↓ 14.62
An02g04680	lipase	↓ 4.93	19.39	4.46	↓ 4.35
An07g04200	triacylglycerol lipase	↓ 3.32	123.82	6.02	↓ 20.57
An18g06580	triacylglycerol lipase	↓ 3.21	58.96	3.63	↓ 16.24
An16g05570	aspartate aminotransferase	↓ 11.93	31.74	1.9	↓ 16.70
An14g01190	arginase	↓ 4.86	37.15	1.4	↓ 26.53
An15g03260	threonine aldolase*	↓ 2.09	9.41	8.47	↓ 1.11
	***Fermentation/glycolysis***				
An02g06820	pyruvate decarboxylase	↓ 17.56	111.28	6.39	↓ 17.41
An08g01520	alcohol dehydrogenase	↓ 194.72	247.83	2	↓ 123.91
An12g09950	alcohol dehydrogenase	↓ 118.28	92.97	0.62	↓ 149.95
An04g02690	alcohol dehydrogenase	↓ 35.46	163.73	21.91	↓ 7.47
An17g01530	*adhA*, alcohol dehydrogenase	↓ 21.35	71.04	5.21	↓ 13.63
An09g03140	alcohol dehydrogenase	↓ 11.59	132.35	3.76	↓ 35.19
An16g05420	glucose-6-phosphate isomerase	↓ 11.61	23.29	2.44	↓ 9.54
An02g14380	*hxkA*, hexokinase	↓ 6.26	24.59	5.16	↓ 4.76
An18g01670	*pfkA*, 6-phosphofructokinase	↓ 64.25	42.44	12.52	↓ 3.38
An02g07470	fructose-bisphosphate aldolase	↓ 22.15	242.31	13.33	↓ 18.17
An08g02260	*pgkA*, phosphoglycerate kinase	↓ 19.01	32.17	15.68	↓ 2.05
An02g03830	*creA*, catabolite repressor	↑ 2.57	4.43	30.22	↑ 6.82
An02g03540	hexose transport protein	↑ 42.48	33.08	523.81	↑ 15.83
	***GABA shunt***				
An10g00090	glutamate dehydrogenase	↓ 89.12	246.31	3.87	↓ 63.64
An15g04770	glutamate decarboxylase	↓ 63.83	62.34	2.3	↓ 27.10
An08g08840	glutamate decarboxylase	↓ 5.74	16.85	3.14	↓ 5.36
An17g00910	GABA transaminase	↓ 4.63	7.63	0	n/a
An14g02870	succinic semialdehyde dehydrogenase	↓ 31.91	274.43	2.34	↓ 117.27
	***TCA cycle***				
An08g05580	isocitrate dehydrogenase	↑ 2.71	10.85	69.17	↑ 6.37
An18g06760	isocitrate dehydrogenase	↑ 3.17	55.23	354.46	↑ 6.41
An04g04750	oxoglutarate dehydrogenase	↑ 2.23	22.9	109.76	↑ 4.79
An17g01670	succinyl-CoA synthetase	↑ 5.36	24.29	128.98	↑ 5.31
	***Metabolism of internal carbohydrates***				
An01g09290	neutral trehalase (*A. nidulans treB*)	↓ 5.01	719.2	58.5	↓ 12.29
An08g10510	*tpsA*, trehalose-6-phosphate synthase	↓ 48.49	124.99	2.69	↓ 46.46
An07g08710	*tpsB*, trehalose-6-phosphate synthase	↓ 2.00	22.52	12.47	↓ 1.80
An11g10990	trehalose-6-phosphate phosphatase	↓ 2.82	73.39	39.34	↓ 1.86
An03g02430	mannitol dehydrogenase	↓ 14.61	74.76	31.38	↓ 2.38
An02g05830	*mpdA*, mannitol-1-phosphate dehydrogenase	↓ 2.96	34.05	6.03	↓ 5.64
An04g04890	glycerol kinase	↓ 24.53	209.95	50.67	↓ 4.14
An08g00210	glycerol-3-phosphate dehydrogenase	↓ 16.76	80.42	25.23	↓ 3.18
An14g04920	*tpiA*, triose-phosphate-isomerase	↓ 29.26	72.81	4.87	↓ 14.95
An16g01830	*gpdA*, glyceraldehyde-3 phosphate dehydrogenase	↓ 14.75	107.99	19.98	↓ 5.40
An07g05790	osmoregulator (*S. cerevisiae SGD1*)	↑ 11.18	3.14	96.62	↑ 30.7
	***Nitrogen metabolism***				
An14g02720	neutral amino acid transporter	↑ 13.44	3.02	313.74	↑ 103.89
An15g07550	neutral amino acid transporter	↑ 2.59	32.67	398.48	↑ 12.20
An16g05880	neutral amino acid transporter	↑ 37.51	14.66	907.29	↑ 61.89
An03g05360	neutral amino acid transporter	↑ 3.97	5.05	127.9	↑ 25.33
An04g09420	neutral amino acid transporter	↑ 58.88	0	126.15	n/a
An17g00860	translation initiation factor (*A. fumigatus cpcC*)	no change^2^	45.91	60.85	↑ 1.32
An01g07900	*cpcA*, transcription factor	↑ 3.55	18.59	123.24	↑ 6.62
An01g08850	transcription factor (*A. nidulans cpcB*)	↑ 3.86	23.52	530.23	↑ 22.54
An11g06180	transcription factor (*A. nidulans prnA*)	↑ 2.59	20.6	101.93	↑ 4.94
An11g06160	proline oxidase (*A. nidulans prnD*)	↑ 5.49	25.16	248.82	↑ 9.88
An11g06150	proline permease (*A. nidulans prnB*)	↑ 2.10	0	9.34	n/a
An11g06140	proline utilisation protein (*A. nidulans prnC*)	↑ 3.67	1.68	70.97	↑ 42.24
An12g08960	*areA*, transcription factor	no change^2^	23.9	12.21	↓ 1.95
An11g00450	nitrate transport protein	↑ 79.28	8.41	909.98	↑ 108.20
An08g05610	*niaD*, nitrate reductase	↑ 5.51	2.19	22.31	↑ 10.18
An08g05640	*niiA*, nitrite reductase*	↑ 2.61	7.34	7.97	↑ 1.08
An18g02330	transcription factor (*A. nidulans nirA*)	present T0, absent T1	45.17	15.7	↓ 2.88
An04g00990	*gdhA*, NADP-dependent glutamate dehydrogenase	↑ 9.61	48.66	1066.53	↑ 21.91
An03g05590	uracil transporter	↑ 28.54	14.71	384.05	↑ 26.11
An11g04340	uracil transporter	↑ 9.43	2.43	28.05	↑ 11.54
An07g01950	uracil transporter (*A. nidulans uapC*)	↓ 8.95	69.95	1.51	↓ 46.32
An01g08050	*uaY,* transcription factor	↓ 5.75	57.87	15.15	↓ 3.82
An14g03370	allantoinase*	↑ 27.48	7.64	16.23	↑ 2.12
	***Mitochondria/respiration***				
An12g01480	Aminoacyl-tRNA biosynthesis	↑ 2.85	20.41	36.35	↑ 1.78
An08g02450	ATP synthase complex assembly	↑ 2.63	4.82	51.51	↑ 10.69
An15g01710	*atp7*, F1Fo-ATP synthase	↑ 2.29	31.59	265.5	↑ 8.40
An01g10880	F1Fo-ATP synthase	↑ 4.53	13.27	427.48	↑ 32.21
An11g04370	cytochrome b5	↑ 65.60	4.32	84.98	↑ 19.67
An02g04330	cytochrome C oxidase	↑ 5.29	0.72	140.73	↑195.46
An08g08720	cytochrome C peroxidase	↑ 11.09	11.03	148.09	↑13.43
An14g00240	holocytochrome-c synthase	↑ 2.50	16.53	48.96	↑ 2.96
An02g12620	mitochondrial respiratory chain complex IV	↑ 5.93	25.57	238.28	↑ 9.32
An08g04150	mitochondrial ribosomal protein	↑ 9.46	7.78	316.39	↑ 40.67
An15g05790	mitochondrial RNA polymerase	↑ 2.56	122.85	236.49	↑ 1.93
An04g02550	mitochondrial translation elongation factor	↑ 33.29	13.97	1080.27	↑ 77.33
An01g10190	mitochondrial transport protein	↑ 41.01	14.25	555.87	↑ 39.01
An08g04240	NADH:ubiquinone reductase	↑ 16.45	4.44	470.4	↑ 105.94
An02g12510	plasma membrane H(+)-ATPase pmaA	↑ 65.65	24.33	1271.65	↑ 52.27
An04g05220	ubiquinol-cytochrome C reductase	↑ 2.95	13.09	213.13	↑ 16.28

Fc^1^ = Fold change based on GeneChip data.

No change^2^ = fold change < 2.

The RPKM values shown are from the combined mapping scores of two distinct biological samples at each time point. Three statistical significance tests were applied to changes in gene expression measured by RNA-seq, the Likelihood Ratio Test [53], Fisher’s Exact Test [54], and an MA-plot-based method with Random Sampling model [52]. All changes in gene transcription, between T0 and T1, listed for RNA-seq data scored a p-value of < 0.001 for all three statistical tests, other than those genes highlighted with a *.

**Figure 2:**
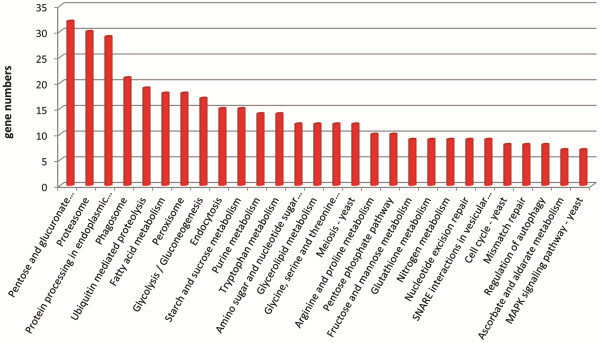
**KEGG categories of down-regulated genes.** KEGG categories of *A. niger* genes having transcripts present at higher abundance in dormant conidia (T0, zero hours) than in conidia germinated for 1 hour (T1) using RNA-seq data.

**Figure 3:**
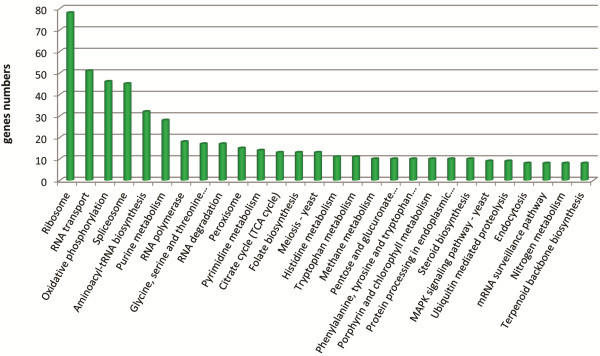
**KEGG categories of up-regulated genes.** KEGG categories of *A. niger* genes showing increased transcript levels at breaking of dormancy (T1, 1 h after germination start) compared to dormancy (T0, zero hours) using RNA-seq data.

To explore transcriptional changes during the first hour of germination in more detail we then used RNA-seq. The RNA-seq results shown are from two separate technical replicates, and we also show the combined mapping scores of those two samples at each time point (Additional file 3). 42.3% (6519 genes) of genes in the combined genome model showed changes (fold-change ≥ 2 using RPKM values) in transcription at T0-T1 which represents approximately 20% more genes than shown by GeneChips. A total of 2626 genes increased their transcript levels and 3893 genes decreased their transcript levels during the first hour of germination. The decreased transcript level gene set was enriched mainly for genes from the KEGG categories of protein degradation, fatty acid metabolism, peroxisome and glycolysis/gluconeogenesis (Figure 2, Additional file 3). The increased transcript level gene set was enriched for the categories of oxidative phosphorylation, RNA processing and protein synthesis (Figure 3, Additional file 3). Gene ontology enrichment analysis was also performed using RNA-seq data and the results are presented in Additional file 4. Amongst genes that were induced at the breaking of dormancy were those encoding functions in cellular metabolic processes reflecting the need of the cell for major metabolic and cellular reorganisations. Protein biosynthesis, nitrogen metabolism and metabolism of RNA represented major functional classes encoded by induced genes. Respiration and mitochondrial metabolism also constituted a large group of functionalities encoded by up-regulated genes suggesting that respiration and functional mitochondria are necessary for germinating conidia. GO enrichment analysis on the down-regulated genes included genes involved in protein degradation, autophagy, carbohydrate metabolism and response to stress.

**Figure 4:**
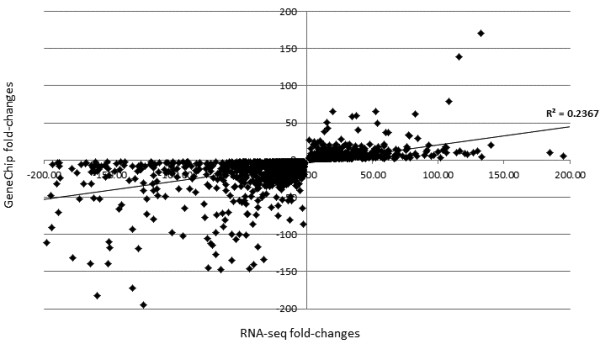
**Correlation of differential gene expression between GeneChips and RNA-seq.** A scatter plot of transcript levels measured with GeneChips and RNA-seq for 5500 genes from developing conidia at T0-T1.

The fold-changes in gene transcription observed either by GeneChips or by RNA-seq showed some correlation (R^2^ = 0.2367) although there were many outliers (Figure 4). The vast majority of genes showed the same pattern of transcription in terms of increased or decreased transcript levels even though the fold-change values varied between the methods for individual transcripts (Additional file 5). Transcript levels measured using RNA-seq have previously been shown to correlate more accurately with protein levels than those measured using microarrays [22].

Dormant conidia showed the most divergent transcript profile in comparison to other examined time points as was also shown by van Leeuwen *et al.*[10] who studied transcriptomes of dormant and germinating (T2-T8) conidia of *A. niger* using Affymetrix GeneChips. We refer throughout to relative transcript levels but we cannot directly infer changes in transcription or turnover of mRNA. Even so, it is highly likely that levels of transcription, and turnover of mRNA, in dormant conidia will be much lower than in germinating conidia. Indeed, the transcripts in dormant conidia may simply be inactive hangovers from the process of conidiation. More interestingly, they may also have some functionality, for example in providing low level maintenance in dormant conidia. Despite these considerations, the major changes in transcripts that are presented, especially during the T0-T1 stage of germination, strongly indicate changes in transcription that support the process of germination. The data presented focus on changes in transcript levels from genes encoding functions related to energy and nitrogen metabolism.

### Transcriptional changes relevant to carbon metabolism

**Figure 5:**
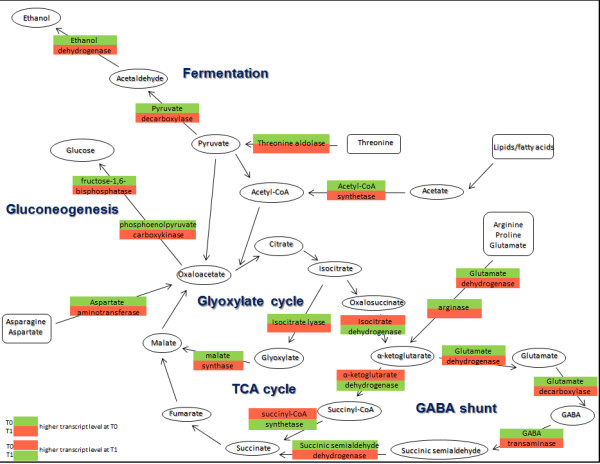
**Model of carbon metabolism.** Selected pathways of carbon metabolism, with an emphasis on the wider aspects of gluconeogenesis, reconstructed from RNA-seq detected changes during the first hour of germination of *A. niger* conidia. Glycolysis is not included but all relevant transcript levels decreased in the transition from T0 (zero hours) to T1 (1 h germination) (Table 1). The green colour represents relatively higher transcript levels in comparison to red colour. The upper color in each rectangle represents dormant conidia (T0), and the bottom colour represents conidia germinated for 1 hour (T1).

An important feature of fungal metabolism is the ability to catabolise a wide range of substrates as carbon sources. Expression of the genes involved in metabolism varies according to the structures of the available substrates. When no preferred carbohydrate is available (e.g. glucose) cells can use alternative sources of energy and change their metabolism accordingly. Our data showed that dormant conidia of *A. niger* contain transcripts of genes encoding enzymes of gluconeogenesis. Gluconeogenesis is a complex metabolic process, whereby the cell can generate glucose from non-carbohydrate carbon substrates when carbohydrates are not available. The transcript levels of key genes involved in gluconeogenesis, such as those encoding fructose-1,6-bisphosphatase (An04g05300, homologous to *A. nidulans acuG*) and phosphoenolpyruvate carboxykinase (An11g02550, homologous to *A. nidulans acuF*) were higher in dormant conidia than in germinated ones (Figure 5).

Lipid-derived fatty acids, acetate and glucogenic amino acids can serve as gluconeogenic substrates. Fatty acids can be degraded via β-oxidation to acetate which, together with the glycerol backbone of membrane and storage lipids, can serve as substrates for gluconeogenesis. Putative lipases which may possibly participate in the degradation of lipids and fatty acids exhibited higher transcript levels in dormant conidia than in T1 germinants. Peroxisomes are organelles where degradation of fatty acids occurs and peroxisomal gene transcripts were present in relatively high abundance in the dormant conidial transcriptome. Acetate in the form of acetyl-CoA is transferred to peroxisomes and mitochondria via acetyl-carnitine and metabolised via the glyoxylate cycle or citric acid cycle, respectively. The transcript level from the putative carnitine O-acetyltransferase gene (An08g04990, *A. nidulans facC*) was lower in T1 germinated conidia compared to that in dormant conidia. Transcripts of An12g01990 and An07g09190 genes encoding putative acyl-CoA synthetases which catalyze the attachment of free fatty acids to coenzyme A in the cytoplasm were more abundant in dormant conidia. The glyoxylate cycle bypasses the steps of the citric acid cycle where carbon is released in the form of CO_2_. It forms an alternative pathway where isocitrate is converted to malate but without production of NADH. Transcripts from genes coding for the enzymes isocitrate lyase (An01g09270, *A. nidulans acuD*) and malate synthase (An15g01860, *A. nidulans acuE*) were more prevalent in dormant conidia than in conidia at T1. Transcript levels of An08g06580 encoding FacB, the transcriptional regulator of acetate metabolism [23] which plays a role in the de-repression of gluconeogenic enzymes [24], were also more highly represented in dormant conidia than in T1 germinants. It has previously been shown that carbon starvation induces conidiation in *A. niger*[25]. When no preferred carbohydrate is present in the environment, cells can use alternative sources of energy and change their metabolism accordingly. Lipids, as potential alternative energy sources, and the presence of isocitrate lyase were detected in dormant *A. fumigatus* conidia [5,26].

Amino acids released from proteins may serve as a free pool of building blocks for new proteins, or as sources of carbon and nitrogen. Several genes that encode enzymes involved in the conversion of glucogenic amino acids into pyruvate or citric acid cycle intermediates had transcripts within the dormant conidia: An15g03260 encodes threonine aldolase that converts threonine to pyruvate, An16g05570 encodes a putative aspartate aminotransferase that may lead to production of oxaloacetate, An02g14590 encodes glutamate dehydrogenase which generates α-ketoglutarate, and An14g01190 encodes arginase which is a component of the arginine catabolic pathway (Figure 5). These products could then serve as precursors for gluconeogenesis.

Transcripts of genes encoding enzymes involved in fermentative metabolism were also detected in dormant conidia. During fermentation, pyruvate is metabolised via acetaldehyde to ethanol. Transcripts from genes coding for putative pyruvate decarboxylases and ethanol dehydrogenases (Table 1) involved in ethanol production were represented in dormant conidia. In dormant conidia of *A. fumigatus*, transcripts and active proteins of fermentative metabolism have previously been reported [1,5]. As mentioned previously, caution is required in interpreting such information because the transcripts detected may be remnants of this metabolic pathway from the process of conidiation.

The GABA shunt bypasses the TCA cycle, contributes to glutamate formation and possibly prevents NADH accumulation in case of limited capacity to use oxygen or when terminal electron acceptors such as oxygen are not available [27]. In *A. nidulans,* this metabolic pathway is active during fermentative growth [28]. Transcripts from genes encoding putative glutamate dehydrogenase (An10g00090), glutamate decarboxylases (An15g04770, An08g08840), GABA transaminase (An17g00910), and succinic semialdehyde dehydrogenase (An14g02870) had relatively high levels in dormant conidia in comparison to T1 germinating conidia (Table 1). In *N. crassa* the activity of glutamate decarboxylase was also present mainly in conidiating structures and conidia and decreased during germination [29]. These data and detection of transcripts of the GABA shunt suggest that this metabolic pathway may be active during conidiation and/or possibly in dormancy.

The transcriptome of T0 dormant conidia was compared with the transcriptome of conidiating *A.niger* cultivated for 6 days in carbon limiting conditions [25]. Metabolic pathways in starved cultures that showed down-regulation of transcription contained genes active in respiration, RNA-processing and translation. Processes that were induced by carbon starvation included fermentation, fatty acid oxidation and amino acid catabolism. Genes involved in gluconeogenesis, glyoxylate cycle and the GABA shunt also showed transcripts during the stages of starvation that were examined. Transcripts of genes playing roles in those pathways were also abundant in the transcriptomes of dormant conidia. Our data, supported by findings in other fungi, imply that fermentation and gluconeogenesis may serve either as an alternative means for replenishing energy during conidiation or may suggest there is some level of metabolism during dormancy, most likely at a very low rate and possibly for maintenance purposes.

The increased energy requirements during germination require increased expression of tricarboxylic acid cycle (TCA cycle) genes. Genes coding for putative isocitrate dehydrogenases (An08g05580, An18g06760), α-ketoglutarate dehydrogenase An04g04750) and succinyl-CoA synthetase (An17g01670) exhibited increased transcript levels at the breaking of dormancy.

After the onset of germination, we detected increased transcript levels of genes encoding putative subunits of the respiratory chain; cytochrome b (An11g04370), cytochrome c oxidase (An02g04330), NADH:ubiquinone reductase (An08g04240) and F1F0 ATPase (An01g10880). Genes encoding proteins involved in the mitochondrial translational machinery and mitochondrial transport also showed increased transcriptional levels mainly during the first hour of germination. Taubitz *et al.*[11] showed that no oxygen was consumed by *A. fumigatus* dormant conidia and that germination is activated only in the presence of oxygen. Although conidiating structures or dormant conidia have access to oxygen, assuming ingress of oxygen through the cell wall, the lack of an easily metabolised substrate such as glucose presumably leads either to a preference for maintenance metabolism through fermentation of non-sugar substrates, or complete dormancy. Our data showed that the transcript levels of these genes were higher in dormant conidia compared to those germinated for 1 h. Upon germination, the switch to aerobic respiration results in a lower rate of glycolysis in *S. cerevisiae*[30] which probably explains the lack of increased transcription of glycolytic genes at breaking of dormancy in *A. niger* conidia.

The availability of glucose is responsible for carbon catabolite repression mediated by the DNA-binding transcriptional repressor CreA which suppresses catabolism of less preferred carbon substrates [31]. As soon as dormant conidia sense enough glucose they up-regulate transcription of the *creA* gene and decrease transcript levels of genes for the glyoxylate cycle and gluconeogenesis during the first hour of germination. Transcription of hexose transporters was shown to be up-regulated at breaking of dormancy (Table 1) which is expected given the necessity of a degradable carbon source for downstream energy production during germination.

### Compatible solutes

**Figure 6:**
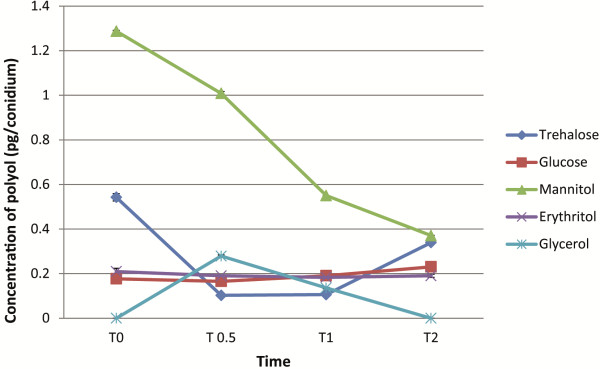
**Changes of internal sugar levels.** Detection of internal polyol levels in dormant *A. niger* conidia and their changes during first two hours (T0 – T2, 0 – 2 h) of germination determined by HPLC. Means and standard deviation of duplicate samples are shown.

Changes in internal sugars during germination have been reported before [6] but this is the first study where their presence was detected and changes were measured over the very early stages of germination. We showed that the switch from catabolism to biosynthesis, especially in the case of mannitol, trehalose and glycerol, occurs during first two hours of germination. We detected trehalose, mannitol, glycerol, erythritol and glucose and measured changes in their levels during the first 2 hours of germination using HPLC (Figure 6) and also analyzed transcription of genes related to their metabolism. In dormant conidia, trehalose, mannitol, erythritol and glucose were detected. Mannitol appeared to be the internal sugar of highest concentration. The breaking of dormancy led to an initial rapid breakdown of trehalose and its re-synthesis shortly afterwards (Figure 6). Mannitol depletion was also initiated at the onset of germination and continued for the first two hours of germination. Its level increased after this time (data not shown). Catabolism of these sugars requires the presence of a carbon source as a trigger in the conidial environment (data not shown). Transcripts of the gene encoding neutral trehalase (An01g09290) involved in trehalose breakdown and trehalose-6-phosphate synthases (*tpsA* An08g10510 and *tpsB* An07g08710) [32] and trehalose-6-phosphate phosphatase (An11g10990) that facilitate trehalose biosynthesis were present at higher levels in dormant conidia. Those levels reduced at the breaking of dormancy and then remained unchanged during the later hours of conidial germination apart from transcripts from the *tpsA* gene which increased over time. Transcripts of genes encoding a putative mannitol dehydrogenase (An03g02430) involved in mannitol catabolism were also found in dormant conidia, as were those coding for a putative enzyme involved in mannitol biosynthesis, mannitol-1-phosphate dehydrogenase (An02g05830). Transcript levels of both of these genes decreased at the breaking of dormancy and remained low throughout germination.

Glycerol metabolism is initiated by the conversion of glycerol to glycerol-3-phosphate (G3P) by glycerol kinase and the G3P is then converted to dihydroxyacetone phosphate by glycerol-3-phosphate dehydrogenase [33]. This is then further metabolised to glyceraldehyde-3-phosphate by triose-phosphate-isomerase. Transcript levels of genes encoding glycerol kinase (An04g04890), glycerol-3-phosphate dehydrogenase (An08g00210), triose-phosphate-isomerase *tpiA* (An14g04920) and glyceraldehyde-3 phosphate dehydrogenase *gpdA* (An16g01830) showed high abundance in dormant conidia and the levels decreased at the breaking of dormancy and did not show up-regulation at later stages of germination. Glycerol wasn’t detected in dormant conidia but its levels reached a peak value after 0.5 h of germination and then dropped below the detection limit at 2 h of germination. Its appearance for a short period of time suggests that conidia undergo major osmotic changes particularly at this time point of germination. An07g05790, a homologue of *S. cerevisiae SGD1* involved in osmoregulatory responses resulting in glycerol production (HOG pathway) [34], increased its transcript level at breaking of dormancy. Contrary to this, Morozova *et al.*[6] detected the presence of glycerol and arabitol in *A. niger* dormant conidia. Erythritol was detected in all examined time points but its level exhibited no significant changes. NADP-dependent erythritol dehydrogenase, involved in the biosynthesis of erythritol, was induced by osmotic stress in *A. oryzae*[14].

Teutschbein *et al*. [5] detected the presence of enzymes responsible for the metabolism of internal solutes in dormant conidia of *A. fumigatus*: a neutral trehalase, mannitol-1-phosphate dehydrogenase and glycerol dehydrogenase. Transcripts of all the genes involved in metabolism of compatible solutes have also been found in conidiating cultures of *A. niger*[25]. Decrease in transcript levels of those genes during germination may suggest readiness of dormant conidia to react promptly in the new environment.

### Protein biosynthesis and nitrogen metabolism in dormant and germinating conidia

One of the most important processes occurring in germinating conidia is synthesis of new proteins. Necessary building blocks for new proteins, amino acids and amino acid precursors, can be recycled or taken up from the environment but the most energy-efficient system is via uptake of free amino acids or amino acid precursors. Our RNA-seq data showed relatively elevated levels of transcripts of amino acid transporter genes over the first hour of germination (Table 1). When the uptake system for amino acids does not result in sufficient supply to fulfil the needs of the growing cell, amino acids have to be synthesized and there are several sensors monitoring the pool of amino acids in the fungal cell. Amino acid starvation is sensed by protein kinase CpcC in *A. fumigatus* (functional homologue of eIF2a kinase Gcn2p in *S. cerevisiae*) [35] and the RNA-seq data showed increased transcript levels from this gene at breaking of dormancy. The signal from CpcC is transmitted to the transcription factor CpcA (An01g07900) (homologue of *S. cerevisiae* Gcn4p), a global regulator in *A. niger* induced by amino acid starvation. Our data showed that transcript levels from *cpcA* increased during the early stage of germination. CpcA regulates transcriptional responses during amino acid starvation by de-repressing the transcription of many genes encoding enzymes involved in amino acid biosynthetic pathways, as well as enzymes involved in nucleotide biosynthesis [36]. An01g08850, a homologue of *A. nidulans cpcB*, represses the transcription of *cpcA* under non-starvation conditions [37]. Its transcript level increased at the breaking of dormancy.

Glutamate, glutamine and ammonia are primary sources of nitrogen for *Aspergillus* spp.. When they are present in very low concentrations, other sources of nitrogen can be used, e.g. nitrate, purines, amino acids, and proteins [38]. Synthesis of specific transporters and enzymes of particular metabolic pathways depends on specificity for a nitrogen substrate present in the environment, and nitrogen catabolite de-repression. AreA is a GATA-type zinc finger transcription factor in *Aspergillus* spp. which activates metabolic pathways of alternative nitrogen sources when primary sources are lacking [39]. In the studies reported here, *A. niger* conidia were produced and germinated in media containing nitrate. The gene cluster responsible for reducing nitrate to ammonia [40] is also present in the *A. niger* genome and ammonia then serves as a source of nitrogen for all amino acids. Genes encoding putative nitrate transporters (e.g. An11g00450) had elevated transcript levels over the course of germination. Genes encoding nitrate reductase *niaD* (An08g05610) and nitrite reductase *niiA* (An08g05640) in the cluster *crnA-niiA-niaD* (Table 1) increased their transcript levels upon germination, but that was not seen with the *crnA* gene (An08g05670) encoding a nitrate transporter. Other studies showed that nitrate signaling only indirectly depends on the CrnA transporter [41] and the *niia* and *niaD* genes are induced by nitrate even in a *crnA*^*-*^ mutant strain [42]. The presence of nitrate in the environment induces their expression and this induction is strictly dependent on the synergistic action of transcription factors NirA (An18g02330) and AreA (An12g08960) [43]. The RNA-seq data showed that transcript levels for both of these genes were higher in dormant conidia. The *gdhA* gene (An04g00990) encoding NADPH-dependent glutamate dehydrogenase exhibited an increased transcript level at breaking of dormancy. This enzyme is required for subsequent incorporation of the ammonium ion. Other studies showed rapid accumulation of mRNA from these genes in *N. crassa* during the presence of nitrate as a sole nitrogen source [44].

It was shown in *A. nidulans* that proline can be used as a source of nitrogen and that there is a cluster of genes responsible for its utilization [45]. This includes the *prnA* gene that encodes the regulatory protein that mediates induction of the whole cluster by proline. *prnD* encodes proline oxidase, *prnB* encodes proline permease, and *prnC* encodes delta-1-pyrroline-5-carboxylate dehydrogenase, the last enzyme in the proline catabolism pathway responsible for its conversion to glutamate. Homologues of those genes are present in the *A. niger* genome (Table 1) and their transcript levels were increased at breaking of dormancy.

*Aspergillus* spp. contain plasma membrane transporters that are specific for the uptake of purine and pyrimidine bases from their growth media [46]. These can be used for nucleotide biosynthesis, and also as nitrogen sources by catabolizing the bases to urea and ammonium. Expression of genes encoding purine-specific transporters and enzymes involved in purine catabolism was repressed in *A. nidulans* by the presence of primary nitrogen sources and induced by purines in the environment [47]. Genes (An03g05590 and An11g04340) encoding putative purine transporters increased their transcript levels at breaking of dormancy. The *uapC* gene (An07g01950) encoding a uric acid/xanthine/purine permease together with the *uaY* gene (An01g08050) [48] encoding a transcriptional regulator of purine utilization, exhibited higher transcript abundance at dormancy than at the first hour of germination. Their transcript levels didn’t exhibit any changes at later stages of germination. Allantoin, the intermediate product of purine metabolism is degraded by allantoinase and the transcript level of gene (An14g03370), encoding a putative allantoinase, was increased at breaking of dormancy.

Based on our data, it seems that inorganic nitrate serves as an efficient nitrogen source for germinating conidia. It is also likely from the transcriptome that germinating conidia hydrolyze proline and purines and use them as nitrogen sources or simply as building blocks in proteins and nucleic acids, respectively.

### Antisense transcription

**Figure 7:**
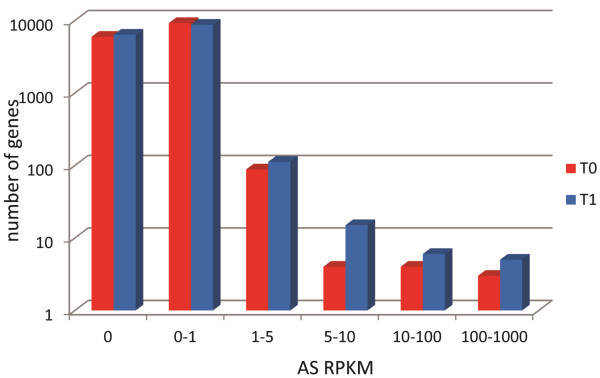
**Distribution of genes with antisense RPKM.** Antisense (AS) RPKM was calculated for each gene in dormant (T0) and germinating (T1) *A. niger* conidia. The number of genes is represented on a log scale.

Antisense transcripts have been identified in various fungi and are transcribed in response to changes in external conditions [21]. Our data showed that the *A. niger* conidial transcriptome also contains natural antisense transcripts (NATs). Antisense (AS) reads from the RNA-seq that fell within the annotated regions of each gene were mapped from both time points (T0 and T1) and antisense RPKM (Reads Per Kilobase of gene model per Million mapped reads) values were calculated. Antisense transcripts represented up to 10% of total gene transcripts in dormant conidia and approximately 5% in T1 germinants, i.e. the majority of genes had very few or no associated antisense transcripts. A total of 100 genes had an AS RPKM greater than 1 and up to about 700 at T0 and 139 genes had an AS RPKM greater than 1 and up to 1100 at T1 (Figure 7, Additional file 6). Antisense transcripts varied in position with respect to their sense transcripts between the entire ORF with upstream and downstream regions to only the 3’ UTR or 5’ UTR.

Transcripts that changed from S to AS or AS to S between examined time points were examined further (Table 2). A total of 13 genes switched from predominant S transcription at T0 to predominant AS transcription at T1. The same genes also showed down-regulation in their sense transcription. This may suggest that down-regulation occurred not only by decreasing sense transcription but also by increasing AS transcription. Examples of genes showing the same transcription pattern were involved in lipid and carbohydrate catabolism, signalling and amino acid metabolism.

**Table 2:** Genes with changed antisense transcription

**(A)**					
**ATCC 1015 ID**	**CBS ID**	**putative gene function**	**T0-T1 fold change ↓**	**T1 AS/T0 AS**	**AS/S at T1**
TID_54223	An18g05740	GTP binding protein	7.96	41.60	5.26
TID_54624	An04g01450	glycolate oxidase	37.69	23.20	3.43
TID_53523	An15g06700	dihydroxy-acid dehydratase	3.98	23.00	0.74*
TID_210938	An14g01050	serine/threonine protein kinase	6.64	19.50	0.46*
TID_57034	An04g03290	acyl-CoA dehydrogenase - β oxidation	2.68	15.50	0.35*
TID_173684	An02g09690	lipase	16.30	15.29	5.04
TID_50444	An04g03950	serine/threonine protein kinase	4.12	13.75	0.75*
TID_197387	An02g06430	transketolase	194.28	11.86	3.91
	An15g02810	phosphatidylinositol phosphate phosphatase	14.05	11.15	36.43
TID_210245	An15g04770	calmodulin-binding glutamate decarboxylase	27.10	7.82	6.26
TID_39560	An07g10430	hypothetical protein involved in stress	6.84	6.95	0.92*
	An01g03400	transcription factor/amino acid metabolism	5.73	6.92	1.30
TID_203198	An12g00030	L-iditol 2-dehydrogenase	12.89	1.76	2.34
**(B)**					
**ATCC 1015 ID**	**CBS ID**	**putative gene function**	**T0-T1 fold change ↑**	**T0 AS/T1 AS**	**AS/S at T0**
TID_57297	An17g02080	hypothetical protein - metal ion transporter	4.36	264.00	0.84*
TID_44497	An03g05020	carnitine/acylcarnitine translocase	1.75	42.38	7.44
TID_187258	An18g03060	leucine carboxyl methyltransferase	14.27	16.86	6.14
TID_52216	An02g04860	cytochrome-b5 reductase	12.36	16.29	5.48
TID_207532	An02g14860	ornithine decarboxylase - polyamine biosynthesis	1.13	12.79	0.92*
TID_182952	An15g01810	hypothetical protein/amino acid metabolism	20.26	9.90	9.99
TID_173423	An02g14950	ATP-dependent RNA helicase	12.04	8.94	0.87*
TID_119526	An06g01130	kinesin heavy chain	3.34	5.68	2.68
TID_46289	An01g13630	oxidoreductase	2.05	3.76	2.21
TID_36448	An01g11750	tyrosine kinase	2.66	3.34	4.43
TID_187212	An18g01610	RNA polymerase II suppressor protein	3.90	2.97	5.42
TID_39426	An11g09660	ethanolaminephosphotransferase	39.61	0.95	11.51
TID_214246	An04g06840	P-type ATPase -Ca2+/phospholipid transport	24.34	−31.00	2.43

* AS/S Ratio is < 1 because part of AS reads do not fall within the ORF but in upstream or downstream regions of these genes therefore were not included in calculations using Ht-seq. Presence of antisense reads in these genes was detected visually using IGV, Integrative Genomic Viewer [49].

(A) Genes with predominant sense transcripts in dormant conidia (T0) that changes to predominant antisense transcription in germinating conidia (T1) showing also down-regulation of sense transcription at T0-T1. (B) Genes with predominant antisense transcription in dormant conidia (T0) that changes to predominant sense transcription in germinating conidia (T1), the same genes also increased their sense transcription at T0-T1. Fold-changes were made using sense and antisense RPKM values generated by RNA-seq.

We have also identified genes that gradually switched from predominant AS transcription at T0 to predominant S transcription at T1. These genes also showed up-regulation in their sense transcription when analysed for differential gene expression (Table 2). Dominant antisense transcription at T0 was enriched in genes involved in transport, RNA-processing and oxidation-reduction reactions.

**Figure 8:**
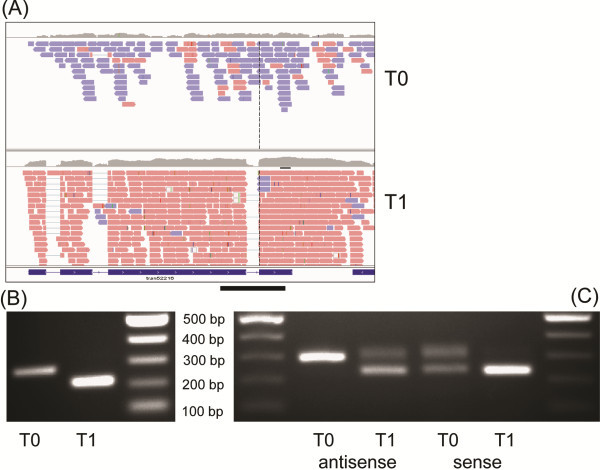
**Sense and antisense transcription of An02g04860.** (**A**) Alignments of sense and antisense reads from two examined time points (T0, T1) as generated by RNA-seq and visualized using the Integrative Genomic Viewer [49]. Blue reads represent antisense transcripts and red reads represent sense transcripts. (**B**) RT-PCR using cDNA as template that was prepared using oligo (dT) primers and amplified using An02g04860 gene-specific primers. The black line in part A represents the amplified region. The size of the non-spliced antisense transcript is 272 bp at T0 and spliced sense transcript is 215 bp at T1. Both PCR products were sequenced to confirm their identities. (**C**) Strand-specific RT-PCR products amplified from cDNA using the tagged primer approach. In dormant conidia (T0), only non-spliced antisense-specific band of high intensity was detected. In germinating conidia both, spliced and non-spliced versions of antisense-specific bands were detected at lower intensity. Fully-spliced sense transcripts of high intensity were detected in germinating conidia (T1) and both spliced and non-spliced transcripts of low intensity were detected in dormant conidia (T0).

In order to confirm the presence of an example NAT, strand-specific RT-PCRs were run for An02g04860 encoding a putative cytochrome-b5 reductase. Figure 8A shows the read alignments for An02g04860 visualised by IGV, Integrative Genomic Viewer [49], and where predominant AS transcription present in dormant conidia changed to S transcription during the first hour of germination. Antisense transcription of three intron regions of this gene was represented whereas the coverage of the sense transcripts in intron areas was very low, indicating that sense transcripts were fully spliced. We presumed that the longer antisense product at T0 switched to the fully spliced sense product by T1. As cDNA was synthesised using oligo (dT) primers, both antisense and sense transcripts were polyadenylated and therefore detected in RT-PCR using primers that bind upstream and downstream of the third intron. cDNA synthesised from mRNA at T0 was detected as a non-spliced product of 272 bp, in contrast to the smaller product corresponding to the fully spliced transcript with a length of 215 bp at T1 (Figure 8B). Both fragments were sequenced and the presence of the 57 bp intron in the larger 272 bp fragment was confirmed. In order to prove the presence of sense/antisense transcripts, strand specific RT-PCRs were performed using a tagged primer approach. Figure 8C shows PCR products amplified from cDNA synthesised specifically from sense or antisense mRNA. Antisense-specific product at T0 was detected only at the larger size, representing the non-spliced version. In germinating conidia there were bands of both sizes suggesting the presence of spliced and non-spliced version of antisense mRNA serving as template. Only fully-spliced sense-specific product of high intensity was detected in germinating conidia. Both spliced and non-spliced sense transcripts of very low intensity were detected in dormant conidia and they may represent true RNA intermediates. These results were in agreement with the data obtained from RNA-seq data showing that a larger antisense transcript predominated in dormant conidia whereas smaller, fully spliced sense transcript was dominant in germinating conidia. Any functional role of antisense transcripts in *A. niger* is not currently understood but, like in other fungi [20], it is possible that antisense transcripts prevent expression of proteins that are not required, i.e. the NATs provide a regulatory control mechanism. Further experiments would be required to confirm their function.

## Conclusions

RNA-seq was used for the first time to uncover transcriptome changes at the breaking of dormancy of *A. niger* conidia. Dormant fungal conidia possess properties that ensure their survival in harsh conditions and they therefore contain protective proteins and their relevant transcripts [2]. Our data showed that the transcriptome of dormant conidia also contains transcripts of genes whose respective proteins were active during conidiation (e.g. carbon starvation genes, genes involved in biosynthesis of mannitol and trehalose) and transcripts of genes necessary for immediate onset of germination (e.g. genes involved in glycerol biosynthesis and catabolism of mannitol and trehalose). Immediate metabolism of internal solutes suggests that conidia are primed for germination. Using RNA-seq methodology the presence of antisense transcripts was shown in dormant conidia and the NATs were represented in higher abundance than in germinating conidia. Antisense transcription was also evident during early germination suggesting that NATs participate in the regulation of changing functionalities at this critical period of conidial outgrowth.

## Methods

### Strains and growth conditions

*A. niger* strain N402, a *cpsA1* derivative of *A. niger* N400 [50] was grown on *Aspergillus* complete medium (ACM) (containing per litre: NaNO_3_ 6 g, KCl, 0.52 g; MgSO_4_.7H_2_O, 0.52 g; KH_2_PO_4_, 1.52 g; Na_2_B_4_O_7_.10H_2_O, 0.008 mg; CuSO_4_.5H_2_O, 0.16 mg; FePO_4_.H_2_O, 0.16 mg; MnSO_4_.4H_2_O, 0.16 mg; NaMoO_4_.2H_2_O, 0.16 mg; ZnSO_4_, 1.6 mg, Bacto casamino acids, 1 g, yeast extract, 1 g, Bacto peptone, 2 g, glucose, 10 g, vitamins: p-aminobenzoic acid, 4 mg, thiamine HCl, 0.5 mg, D-biotin, 0.02 mg, nicotinic acid, 1 mg, pyridoxine hydrochloride, 2.5 mg, choline chloride, 0.014 g, riboflavin, 1 mg, agar 20 g where applicable) for 6 days at 28°C to develop mature conidia. Conidia were harvested by washing the agar slopes with a 0.01% (w/v) Tween 80 solution. The conidial suspension was filtered through sterile synthetic wool and conidia were counted using a haemocytometer.

### RNA extraction

Dormant *A. niger* conidia were harvested from ACM slopes incubated for 6 days. Conidia (10^4^/ml) were germinated in liquid ACM media for 1, 2, 4 and 6 hours at 28°C, in 2 L conical flasks containing 1000 ml of medium, shaken at 150 rpm. Germinated conidia were recovered by filtration into 0.5 ml RNA extraction buffer (0.6 M NaCl, 0.2 M sodium acetate, 0.1 M EDTA, 4% w/v SDS) and snap frozen in liquid nitrogen. Frozen dormant or germinated conidia were mixed with 0.5 ml glass beads and disintegrated in a Sartorius dismembranator (4 min, 2000 rpm).

For GeneChip studies, RNA was extracted using the TRIzol reagent protocol (Invitrogen) according to manufacturer’s instructions, followed by an additional clean-up using RNeasy columns (Qiagen) including the on-column DNAase treatment step. RNA for each individual experiment (time point) contained pooled RNAs (10 μg) from three independent RNA extractions and only 1 technical replicate for each time point was used. Quality checks and subsequent GeneChip experiments were performed at The Nottingham *Arabidopsis* Stock Centre (NASC, University of Nottingham, Sutton Bonington Campus, UK), using *A. niger* GeneChips provided by Affymetrix and supplied by DSM [17].

RNA for RNA-seq experiments also contained pooled RNAs from three independent RNA extractions and 2 technical replicate for each time point were used. Samples were purified after dismembranation using the Plant/Fungi total RNA Purification Kit (Norgen Biotek, Canada) including the on-column DNAase treatment step. The concentration and quality of RNA for each sample was determined by UV spectrometry (Nanodrop ND-1000 spectrophotometer). Quality checks and subsequent RNA-seq experiments were performed at the Next Generation Sequencing Facility (Queen's Medical Centre, University of Nottingham, UK).

### cDNA labelling, hybridisation and analysis of Gene Chip data

Standard Affymetrix eukaryotic target sample preparations and hybridisation protocols were followed as described in the Affymetrix technical manual (http://www.affymetrix.com) and performed at European Arabidopsis Stock Centre (NASC). The RNA integrity of each sample was determined using an Agilent 2100 Bioanalyzer™ (Agilent). *A. niger* GeneChips were hybridised, washed, stained and scanned according to the Affymetrix protocols (http://www.affymetrix.com). Array descriptions/probe IDs were aligned to gene accession numbers [17]. Affymetrix Expression Console™ generated CHP.files and showed the total numbers of present, marginal and absent detection calls from each experiment. Raw data were analysed using the software GeneSpring GX 11 (Agilent Technologies, Inc). They were normalized using the RMA (Robust Multichip Analysis) global normalization algorithm. Raw intensity signal values were normalized per chip to the 75^th^ percentile and baseline transformation to the median of all samples (time points) was used. Raw data files have been submitted to the Gene Expression Omnibus, under accession number (GSE42480). To predict the cellular functions associated with the observed changes in transcript levels, genes with fold-change ≥ 2 (as generated by GeneSpring) were categorized according to predicted protein function using the Kyoto Encyclopedia of Genes and Genomes (KEGG) database (http://www.genome.jp/kegg/).

### RNA-seq methodology and data analysis

10 μg of Total RNA was depleted of ribosomal RNA using the Ribominus Eukaryotic kit (Invitrogen). SOLiD whole transcriptome libraries were made as outlined in the SOLiD Total RNA-Seq kit protocol (Applied Biosystems). Libraries were quantified by qPCR using a KAPA library quantification kit for Applied Biosystems SOLiD platform and pooled in equimolar amounts. Pooled libraries were gel-purified using 2% size-select E-gels to 200-300 bp (Invitrogen). Emulsion PCR and bead-based enrichment was carried out using the SOLiD EZ bead system. Sequencing was performed on a SOLiD 5500xl ABi sequencer according to the manufacturer’s instructions to generate 50 bp/35 bp paired-end reads in colour space.

Reads were mapped to the genome sequence assembly of the *A. niger* ATCC 1015 strain as it is the most closely related sequenced strain to the N402 strain used in this study. In order to ensure the most comprehensive gene model possible, genes that are predicted in the CBS 513.88 genome, but absent in the ATCC 1015 model, were mapped to the *A. niger* JGIv3 Genome sequence using GMAP and Exonerate. GMAP: all selected Ensembl gene cDNA sequences were aligned to the genome (default settings). Exonerate: all selected Ensembl gene PROTEIN sequences were aligned to the genome with exonerate2protein (default settings). All GMAP alignment results were accepted first. Those not mapped by GMAP, but mapped by exonerate were then integrated into the annotation. SOLiD reads were mapped and read counts per gene were determined using the LifeScope 2.5.1 Whole Transcriptome Pipeline (LifeTechnologies). Reads were initially filtered against sequencing adaptors and barcodes and a collection of published *A. niger* rRNA sequences prior to read mapping. LifeScope provided all read alignment positions of each paired-read mapped against the complete genome sequence and exon spanning junctions using the GTF gene annotation information. Read alignment results were recorded in BAM format for further downstream analysis. Read counts per gene were determined from primary read alignments with a mapping quality of 20 or more (MAPQ20). These counts were then used to calculate normalized expression values (RPKM) (Reads Per Kilobase of gene model per Million mapped reads) for each gene [51] as well as being the input for determining significant differential gene expression. Antisense transcription was detected by comparing gene counts generated by Htseq-count (http://www-huber.embl.de/users/anders/HTSeq) using F3. Bam files as input and opting to ignore or include strand-specificity in the calculations. Data were visualised using IGV, Integrative Genomic Viewer [49].

Differential gene expression was analysed using the R package DEGseq [52]. Three statistical significance tests were applied to changes in gene transcription, the Likelihood Ratio Test [53], Fisher’s Exact Test [54], and an MA-plot-based method with Random Sampling model [52]. To predict the cellular and metabolic functions associated with the observed changes in transcript levels, genes with fold-change ≥ 2 using RPKM values were categorized according to predicted protein function using the Kyoto Encyclopedia of Genes and Genomes (KEGG) database (http://www.genome.jp/kegg/). GO enrichment analysis was also performed using the set of differentially expressed genes (FetGOat: http://www.broadinstitute.org/fetgoat/index.html) that had RPKM fold-change ≥ 2 at T0-T1. Data files have been submitted to the Gene Expression Omnibus, under accession number (GSE42652).

### Internal stores of carbohydrates

#### Extraction of cytosolic carbohydrates

10^8^ dormant or germinating conidia were collected by centrifugation (11,000×g, 5 minutes), washed three times with 10 ml sterile water, re-suspended in 1 ml 0.25 M Na_2_CO_3_ and subjected to mechanical disruption using the dismembranator as previously described. Samples were centrifuged (11,000×g, 5 minutes) and 500 μl of the supernatants were filtered through 0.2 μm filters (Sartorius Stedim Biotech, Germany) for HPLC analysis of carbohydrate content.

#### HPLC determination of polyols

Standard compounds for analysis were obtained from Sigma unless otherwise stated. The compounds studied - mannitol, trehalose, erythritol, glucose (Fisher Scientific UK Limited) and glycerol (Courtin and Warner Ltd) were used as standards. Polyols present in the samples were analyzed by HPLC (Agilent technologies 1200 series). Samples (20 μl) were applied to an ion exclusion column (Aminex HPX-87H, 7.8×300 mm, Bio-Rad Laboratories Inc, Hertfordshire, UK) at 60°C, using an isocratic elution with 0.01 N H_2_SO_4_ at 0.6 ml min^-1^. Detection was carried out using a refractive index detector. Each compound was run on the column to determine the retention time in minutes. A calibration was carried out for each compound and concentration of each compound was plotted against peak area. The concentrations of sugars in sample were calculated using calibration curves. Cytosolic extracts obtained from dormant conidia (0 h) and germinating conidia over a 2 h period (0.5 h, 1 h and 2 h) were analyzed in duplicate.

### RT-PCR and strand specific RT-PCR

Total RNAs SuperScript™ III reverse transcriptase (Invitrogen) was used to prepare cDNA from total RNA according to manufacturer’s instructions using oligo (dT) as primers and amplified using gene specific primers, CYT-Forward and CYT-Reverse. Specific sequences were added at the 5’ ends of the original RT-PCR primer pair and these tagged gene-specific primers (CYT-tag-S, CYT-tag-AS) were used to specifically transcribe cDNA from sense and antisense RNA strand in strand specific reverse transcription. Using primer identical to the added tag sequence (CYT-tag-F, CYT-tag-R) together with opposing gene-specific primer (CYT-Forward, CYT-Reverse) ensured that only cDNA synthesised from the tagged primer was amplified. 1 μg of total RNA was used in each reverse transcription reaction. PCR reactions were performed using Phusion polymerase (New England Biolabs) in 50 μl reactions, 98°C for 4 min followed by 35 cycles: 98°C 30 s, 56°C 30 s and 72°C 30 s. Primers used for cDNA synthesis, RT-PCRs and strand specific RT-PCRs are listed in Additional file 7.

## Competing interests

The authors declare that they have no competing interests.

## Authors’ contributions

MN and KH prepared samples for Affymetrix GeneChips and conducted data analysis. MN prepared samples for RNA-seq, performed data analysis, accomplished antisense detection study and drafted the manuscript. KH performed HPLC analysis of internal solutes. MB and RW carried out RNA-seq experiment and data assembly. SP helped with GeneChip and RNA-seq bioinformatic analysis. DA, MS and MN designed the study, contributed to discussion and finalized the manuscript. HS provided Affymetrix GeneChips and contributed to discussion. All authors read and approved the final manuscript.

## Supplementary Material

Additional file 1: Figure S1 Percentage of detected calls. Percentage of Affymetrix probe sets having A = absent, M = marginal, or P = present detection calls at all examined time points (T0 – T6, in hours). *A. niger* conidia developed over time and extracted RNA was used to probe the GeneChips. Click here for file

Additional file 2 **Differentially expressed genes at T0-T6.** Fold-changes of transcript levels from GeneChips between examined time points (T0-T1, T1-T2, T2-T4, T4-T6) as generated by GeneSpring using CEL.files as inputs, and groupings into KEGG categories. Click here for file

Additional file 3 **Expression values and KEGG analysis of differentially expressed genes.** Single and combined mapping scores and RPKM values for all genes in dormant (T0) and germinating (T1) conidia, statistical significance values calculated using R package DEGseq [52] and, categorization according to the KEGG database. Click here for file

Additional file 4 **GO analysis of differentially expressed genes.** Gene ontology categories in up-regulated and down-regulated groups of genes (FetGOat: http://www.broadinstitute.org/fetgoat/index.html) using RNA-seq data. Click here for file

Additional file 5 **Differentially expressed genes at T0-T1.** Fold-changes of transcript levels from RNA-seq and GeneChips compared side by side. Click here for file

Additional file 6 **Antisense and sense transcription profiles.** Antisense and sense RPKM values and their ratios in dormant (T0) and germinating (T1) conidia for all the genes with detected AS transcription at any time generated by Ht-seq. Click here for file

Additional file 7 **Primers used in this study.** Primers and their sequences used for strand-specific cDNA synthesis, RT-PCRs and strand-specific RT-PCRs. Click here for file
