# Supplementary figures and images for: Trancriptional landscape of Aspergillus niger at breaking of conidial dormancy revealed by RNA-sequencing
Source: BMC Genomics. 2013 Apr 11;14:246. doi: 10.1186/1471-2164-14-246 (PMC3635940; doi:10.1186/1471-2164-14-246)

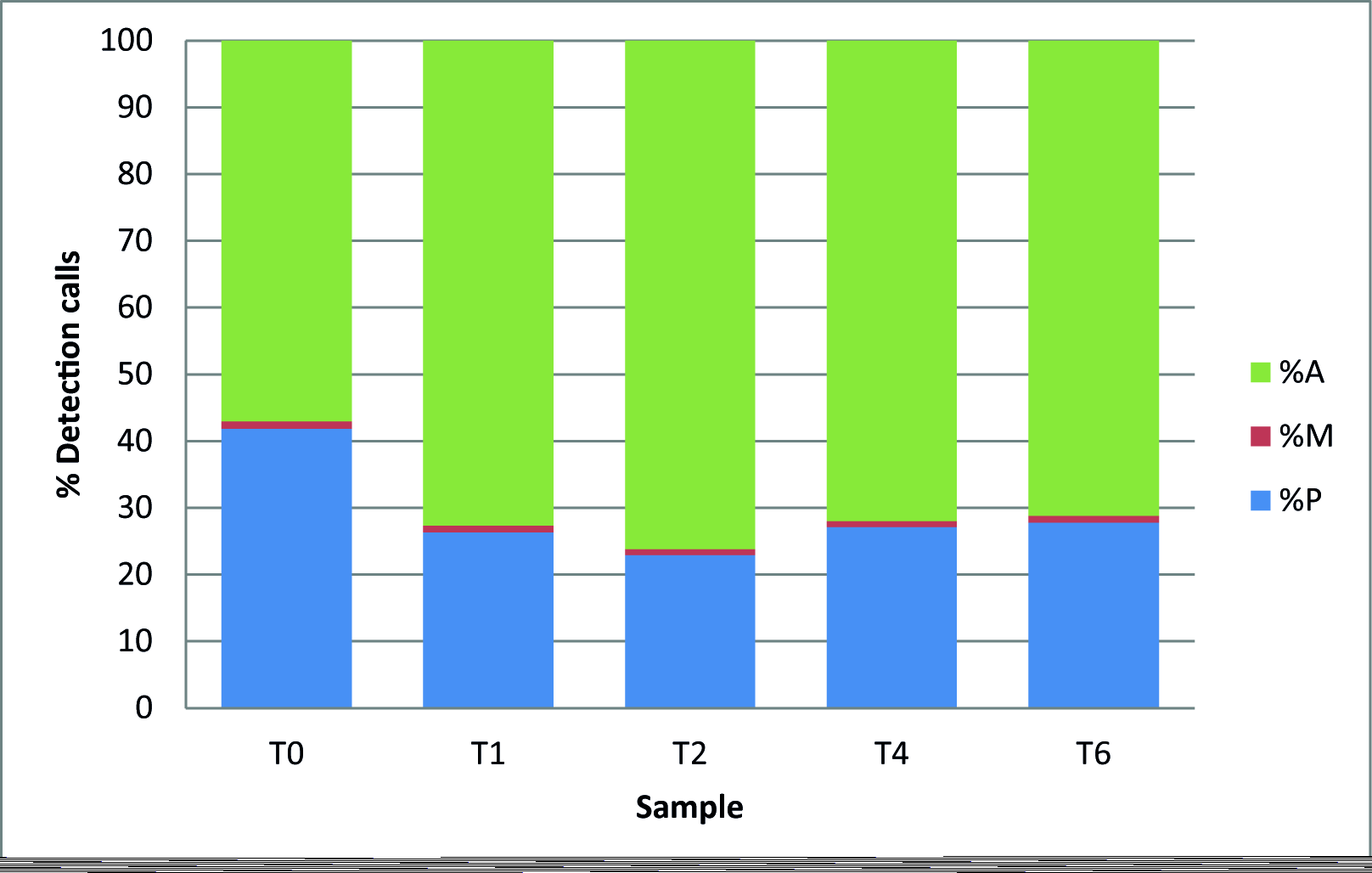

Supplement: Additional file 1: Figure S1 — Percentage of detected calls. Percentage of Affymetrix probe sets having A = absent, M = marginal, or P = present detection calls at all examined time points (T0 – T6, in hours). A. niger conidia developed over time and extracted RNA was used to probe the GeneChips. [file 1471-2164-14-246-S1.tiff]
